# A systematic review of the effects of e-cigarette use on lung function

**DOI:** 10.1038/s41533-022-00311-w

**Published:** 2022-10-22

**Authors:** Lucy Honeycutt, Katherine Huerne, Alanna Miller, Erica Wennberg, Kristian B. Filion, Roland Grad, Andrea S. Gershon, Carolyn Ells, Genevieve Gore, Andrea Benedetti, Brett Thombs, Mark J. Eisenberg

**Affiliations:** 1 Lady Davis Institute for Medical Research, Jewish General Hospital/McGill University, Montreal, QC Canada; 2 Biomedical Ethics Unit, Departments of Medicine and Social Studies of Medicine, and Division of Experimental Medicine, McGill University, Montreal, QC Canada; 3 Departments of Medicine and of Epidemiology, Biostatistics and Occupational Health, McGill University, Montreal, QC Canada; 4 Department of Family Medicine, McGill University, Montreal, QC Canada; 5 Division of Respirology, Department of Medicine, Sunnybrook Health Sciences Centre and the University of Toronto, Toronto, ON Canada; 6 Schulich Library of Physical Sciences, Life Sciences, and Engineering, McGill University, Montreal, QC Canada; 7 Respiratory Epidemiology and Clinical Research Unit, Centre for Outcomes Research and Evaluation, Research Institute of the McGill University Health Centre, Montreal, QC Canada; 8 Departments of Psychiatry, Psychology, and Biomedical Ethics Unit, McGill University, Montreal, QC Canada; 9 Division of Cardiology, Jewish General Hospital/McGill University, Montreal, QC Canada

**Keywords:** Epidemiology, Epidemiology

## Abstract

Given the increasing use of e-cigarettes and uncertainty surrounding their safety, we conducted a systematic review to determine the effects of e-cigarettes on measures of lung function. We systematically searched EMBASE, MEDLINE, and PsycINFO databases via Ovid, the Cochrane CENTRAL database, and the Web of Science Core from 2004 until July 2021, identifying 8856 potentially eligible studies. A total of eight studies (seven studying immediate effects and one long-term effects, 273 total participants) were included. The risk of bias was assessed using the Risk of Bias in Non-randomized Studies—of Interventions (ROBINS-I) and Cochrane risk of bias tools. These studies suggest that vaping increases airway resistance but does not appear to impact forced expiratory volume in one second (FEV_1)_, forced vital capacity (FVC), or FEV_1_/FVC ratio. However, given the limited size and follow-up duration of these studies, larger, long-term studies are required to further determine the effects of e-cigarettes on lung function.

## Introduction

The first electronic cigarette (e-cigarette) was patented and marketed in 2004^1^. Since then, e-cigarette use (or “vaping”) has grown exponentially across the globe^2^. As the use of vaping devices evolves with policy, the consequences of vaping on health are becoming an increasingly important public health issue. E-cigarettes are being studied for harm reduction in individuals who use cigarettes and as a smoking cessation aid, as they are believed to be less harmful to health than smoking^3^. However, there is increasing evidence demonstrating adverse respiratory effects of vaping compared to vaping abstinence. In particular, an outbreak of E-Cigarette and Vaping-Associated Lung Illness (EVALI) brought the short-term respiratory consequences of vaping into question, especially if cannabis or THC-containing products are used^4^. Other short-term respiratory changes that have been linked to vaping include increased airway resistance^5^, breathing difficulty^6^, and transient lung inflammation^7^. Vaping has also been associated with chronic respiratory conditions such as asthma^8^ and chronic bronchitis^9^. Despite these reports, the short- and long-term respiratory safety of vaping is still largely unknown. Several small studies have examined the effects of e-cigarettes on lung function, including measures such as forced expiratory volume in one second (FEV_1_), forced vital capacity (FVC), and airway resistance. However, no evidence syntheses have been completed on this topic. Therefore, we conducted a systematic review to determine the effects of vaping on measures of lung function.

## Methods

Our systematic review was conducted following a protocol developed prior to initiating the review, which was registered on the PROSPERO register of systematic reviews (CRD42021227121)^10^. This systematic review is reported following the Preferred Reporting Items for Systematic Reviews and Meta-Analyses (PRISMA) guidelines^11^.

### Search strategy and study selection

Using a search strategy (Supplementary Tables 1–5) developed by an experienced health sciences librarian (G.G.), we systematically searched EMBASE, MEDLINE, and PsycINFO databases via Ovid, the Cochrane CENTRAL database, and the Web of Science Core from 2004 (the year of the first e-cigarette patent) until July 12, 2021. We additionally conducted a gray literature search by searching the websites of key governmental and public health organizations (the World Health Organization, Health Canada, the US Centers for Disease Control and Prevention, the US Food and Drug Administration, the Canadian Center on Substance Use and Addiction, the European Centre for Disease Prevention and Control, and the European Public Health Association). Additional articles were identified by manually searching the reference lists of included publications as well as SCOPUS and Google Scholar (first ten pages). Articles were included if they reported quantitative primary data on changes in lung function associated with vaping, defined as the use of any device that functions by transforming an e-liquid to an aerosol using metal coils, among human participants of any age. Studies of cells and those conducted in animals were excluded. Studies using heat-not-burn devices were also excluded, as these do not meet the above definition of vaping. Eligible studies included randomized controlled trials (RCTs), non-randomized studies of interventions (NRSIs), and cohort studies; cross-sectional studies and case reports were excluded. We included studies that used non-users of both vaping devices and conventional cigarettes as a comparison group and those that used a pre- and post-design in which individuals acted as their own controls. Inclusion was not restricted by language or country of publication. Abstracts and conference proceedings were included if sufficient data could be extracted from these publications.

Search results were downloaded from databases into reference management software (EndNote X9) or manually added (e.g., for gray literature results). Duplicates were removed in EndNote and entries were uploaded to Covidence (Veritas Health Innovation, Melbourne, Australia), a systematic review software. Two reviewers (L.H. and K.H.) independently screened the titles and abstracts of all identified publications for eligibility. Citations considered potentially eligible by either reviewer, based on the pre-specified review inclusion/exclusion criteria (Supplementary Table 6), were retrieved for full-text screening and assessed for inclusion. The reasons for exclusion after full-text review were annotated in Covidence and any disagreements were resolved by consensus or a third reviewer (A.H-L.).

### Data extraction

Two independent reviewers (L.H and K.H.) extracted methodological, demographic, and outcome data from included studies in duplicate; disagreements were detected in Covidence and were resolved by consensus or, if necessary, by a third reviewer (A.H-L.). Extracted data included study characteristics (first author, journal, year of publication, years(s) of data collection, funding, data source, study design, recruitment strategy, duration of follow-up, country of origin, sample size); population characteristics (sex, gender, age, race, ethnicity, socioeconomic status, dose/frequency of e-cigarette use, conventional cigarette smoking status, smoked cannabis use); and vaping behavior, including the type of vaping device used (e.g., disposable e-cigarette vs. pod device such as JUUL), vaping products used (e.g., nicotine cartridges exclusively vs. THC cartridges exclusively vs. dual use of nicotine and THC products), and source of the vaping product (informal [i.e., friends, family members, or dealers] vs. commercial [i.e., vape shops, stores, dispensaries]).

Initially, extracted outcomes of primary interest were respiratory signs and symptoms, as these are important to patients and are the early signs of respiratory disease. Secondary outcomes included: findings on lung function; Computed tomography (CT) findings of emphysema, airway remodeling, and small airway loss; respiratory-related quality of life and exercise limitations; incidence and/or prevalence of respiratory disease as well as exacerbations of previous respiratory disease; and health care resource use including respiratory disease-related ambulatory care, emergency department visits, and hospitalization. Given the limited number of studies available and the heterogeneity of the data extracted from these studies, no meta-analysis was conducted.

### Risk of bias

The risk of bias in included publications was assessed independently by two reviewers (L.H. and K.H.), and discrepancies were resolved by consensus or, if necessary, by a third reviewer (A.H-L.). The risk of bias of included non-randomized studies (pre-post studies, NRSI with non-vaping reference group, cohort study) was assessed using the Risk of Bias in Non-randomized Studies—of Interventions (ROBINS-I) tool^12^. The ROBINS-I tool evaluates intervention-specific outcomes for a study through seven domains which assess the risk of bias pre-intervention, at-intervention, and post-intervention. For each outcome of interest extracted from an included study, the risk of bias within each domain was reported as “low”, “moderate”, “serious”, or “critical”. Included RCTs were assessed using the Cochrane Collaboration’s Tool for Assessing Risk of Bias (ROB V1)^13^. Similar to ROBINS-I, this tool evaluates the risk of bias through the assessment of five domains; for each outcome of interest extracted from an included study, the risk of bias for each domain was reported as “low risk of bias”, “high risk of bias”, or “unclear risk of bias.” All eligible publications were included in the qualitative synthesis regardless of their assessed risk of bias.

### Reporting summary

Further information on research design is available in the Nature Research Reporting Summary linked to this article.

## Results

**Fig. 1:**
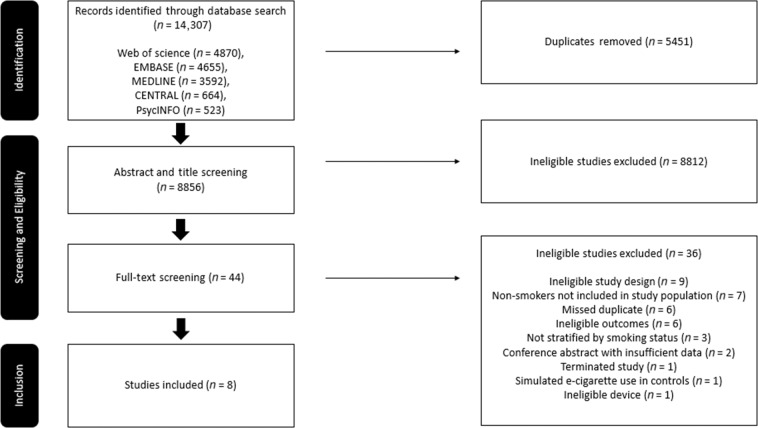
PRISMA flow diagram of included studies assessing the effect of e-cigarettes on lung function.

As our search did not identify studies which focused on the broad outcomes detailed above, we chose to limit our focus to studies on lung function. Our database searches identified 14,307 potentially eligible studies (Fig. 1). After duplicates were removed, 8856 titles and abstracts were assessed. After this initial screening, 44 full texts were retrieved and reviewed in further detail, yielding eight studies eligible for inclusion.

### Study and participant characteristics

Of the eight included studies (273 total participants), seven^14–20^ involved short-term exposure to e-cigarettes with immediate outcome assessment, and the remaining study followed vapers and non-vapers over 3.5 years^21^ (Table 1). This prospective cohort study examined 21 participants (12 nonsmokers and nine vapers) at means of 12 (standard deviation: 1), 24 (2), and 42 (2) months after baseline^21^. Of the seven short-term studies, four were NRSIs (three pre-post studies^14–16^ and one NRSI with a non-vaping reference group^20^) and three were RCTs^17–19^. Among these seven studies, two included 70–80 participants^14,15^ and five included 10–30 participants^16–20^. Exposures varied in terms of e-cigarettes, e-liquids, and vaping session timings. Most studies did not expand on their definition of “non-smoker/non-vaper”^15,16,18–21^, but two studies clarified that these participants were never-smokers^14,17^. One of these two studies further elaborated that participants had no exposure to tobacco products or e-cigarettes^17^. Few studies gave detailed information on the type of e-cigarette used. Three studies reported a specific brand or product (Blu^17^, eGo^16^, Joytech elips-C series^18^, Puff bar^20^). Polosa et al. listed some of the various e-cigarettes used by participants throughout the longitudinal study, including standard refillable (eGo style products) and more advanced refillable (Provari, Innokin, Joytech, eVIC, Avatar Puff)^21^. The remaining studies did not report a specific brand, though one study described the e-cigarette used as a “1^st^ generation e-cigarette popular in Greece”^15^. All studies clarified whether the e-cigarettes used during the study contained nicotine.

**Table 1:** Characteristics of studies examining the effects of e-cigarettes on lung function.

Study	Location	Design	Intervention/Exposure and timing	Comparator	Sample size	Participant population	Outcomes of interest
Short-term studies							
Palamidas 2017	Greece	NRSI (pre-post)	10 min vaping with EC	Smokers: none Non-smokers: 11 mg or 0 mg nicotine	76	55 smokers: 16 COPD: 12/16 male, 61 ± 9 years; 11 asthma: 4/11 male, 37 ± 10 years; 28 no respiratory history: 16/28 male, 41 ± 10 years 21 healthy nonsmokers: 9 in 11 mg group: 5/9 male,35 ± 13 years; 12 in 0 mg group: 7/12 male, 34 ± 10 years	FEV_1_, FVC, FEV_1_/FVC, airway resistance, specific airway conductance, oxygen saturation
Palamidas 2013	Greece	NRSI (pre-post)	10 min vaping with EC	Smokers: none Non-smokers: 11 mg or 0 mg nicotine	70	Group A (nicotine e-cig): nine never-smokers and 51 smokers (24 with no overt airway disease, 11 asthma, 16 COPD) Group B (nicotine-free e-cig): 10 never-smokers	airway resistance, specific airway conductance
Coppeta 2018	Italy	NRSI (pre-post)	EC and CC, 15-min sessions on different days (15 puffs EC)	EC or CC	30	30 nonsmokers: 17/30 male, 32.6 ± 2.75 years	FEF_25-75_, FEV_1_, FEV_1_/FVC
Kizhakke Puliyakote 2021	USA (CA)	NRSI (non-vaping reference group)	EC, unspecified duration (only in baseline vapers)	non-vapers or vapers	16	9 vapers: 6/9 male, 21 ± 2 years Seven nonsmokers: 4/7 male, 23 ± 5 years	FEV_1_, FEV_1_/FVC, FVC, oxygen saturation
Ferrari 2015	Italy	RCT	CC and nicotine-free EC ad libitum for 5 minutes in two different sessions	EC or CC	20	Ten smokers: 4/10 male, 42.3 ± 12.6 years Ten nonsmokers: 7/10 male, 36.2 ± 12.3 years	FEF_25_, FEF_50_, FEF_75_, FEV_1_, FEV_1_/FVC, FVC
Boulay 2017	Canada	RCT	Three inhalations of EC per minute for 1 h; 2 × 1-h sessions 1 week apart	none	30	30 subjects, all nonsmokers: 20 healthy (age 20–37 years); ten asthmatic (age 21–40 years)	FEV_1_, FEV_1_/FVC, FVC, oxygen saturation
Staudt 2018	USA (NY)	RCT	Two sessions 30 min apart, ten puffs EC	nicotine or non-nicotine	10	Nicotine group, seven nonsmokers: 4/7 male, 40.4 ± 11.2 years Non-nicotine group, three nonsmokers: 1/3 male, 39.7 ± 6.7 years	FEV_1_, FEV_1_/FVC, FVC, oxygen saturation
Long-term studies							
Polosa 2017	Italy	NRSI (cohort study)	3.5 year follow-up of nonsmokers and vapers at 12 (±1), 24 (±2), and 42 (±2) months after baseline visits	none	21	9 vapers: 6/9 male, 26.6 ± 6.0 years 12 nonsmokers: 8/12 male, 27.8 ± 5.2 years	FEF_25_, FEF_25-75_, FEV_1_, FEV_1_/FVC, FVC

*CC* conventional cigarette, *COPD* chronic obstructive pulmonary disease, *EC* electronic cigarette, *FEF*_25_ forced expiratory flow 25%, *FEF*_50_ forced expiratory flow 50%, *FEF*_75_ forced expiratory flow 75%, *FEF*_25-75_ maximum mid-expiratory flow, *FEV*_1_ forced expiratory volume in one second, *NRSI* non-randomized study of intervention, RCT randomized controlled trial.

### Risk of bias

The included RCTs (*n* = 3)^17–19^ had an unclear risk of bias, with each study demonstrating an unclear risk of bias in 3+ domains (Table 2). This was primarily due to missing information in the manuscripts required to make an adequate judgment, such as a lack of detail surrounding randomization. The risk associated with the blinding of participants and personnel was judged to be low for all 3 included RCTs. These studies were not blinded, and one was placebo-controlled. However, it was judged that this lack of blinding would not influence measures of lung function. Of the included non-randomized studies (*n* = 5)^14–16,20,21^, four^14–16,20^ were judged to be at moderate risk of bias and one^21^ was found to have a serious risk of bias (Table 3). The most consistent source of bias in these studies was bias due to confounding, with only one^16^ study judged to have a low risk of bias due to confounding. Of the remaining four studies, three^14,15,20^ were found to have a moderate risk of bias due to confounding and one^21^ was found to be at serious risk of bias due to confounding, with important confounding variables not accounted for in the design or analysis.

**Table 2:** Quality assessment of randomized controlled trials examining the effects of e-cigarettes on lung function, as defined by the Cochrane Collaboration Risk of Bias tool (version 1).

	Random sequence generation	Allocation concealment	Blinding of participants and personnel	Blinding of outcome assessment	Incomplete outcome data	Selective reporting	Other bias
Ferrari 2015	Unclear	Unclear	Low	Low	Unclear	Low	High
Boulay 2017	Unclear	Unclear	Low	Unclear	Low	Unclear	Unclear
Staudt 2018	Unclear	Unclear	Low	Unclear	Low	Low	Low

**Table 3:** Quality assessment of non-randomized studies of interventions examining the effects of e-cigarettes on lung function, as defined by the ROBINS-I tool.

	Bias due to confounding	Bias in the selection of participants for the study	Bias in the classification of interventions	Bias due to deviations from the intended intervention	Bias due to missing data	Bias in the measurement of outcomes	Bias in the selection of the reported result	Overall
Palamidas 2013	Moderate	Moderate	Low	Low	No Information	No Information	Moderate	Moderate
Palamidas 2017	Moderate	Low	Low	Low	Low	No Information	Low	Moderate
Polosa 2017	Serious	Low	Low	Low	Moderate	Low	Moderate	Serious
Coppeta 2018	Low	Low	Low	Low	No Information	Moderate	Low	Moderate
Kizhakke Puliyakote 2021	Moderate	Low	Moderate	Low	Low	Moderate	Low	Moderate

### Effects of E-cigarette use on lung function

Seven studies^14–20^ reported immediate measures of lung function after short-term exposure to e-cigarettes (Table 4), including FEV_1_, FVC, and FEV_1_/FVC. Two studies, Boulay et al. and Staudt. et al. suggested no changes in FEV_1_ or FEV_1_/FVC after vaping among nonsmokers^17,19^. Kizhakke Puliyakote et al. observed lower baseline FEV_1_ and FEV_1_/FVC values among nonsmokers compared to vapers^20^. Coppeta et al. found a decrease in FEV_1_ and FEV_1_/FVC among nonsmokers after 1 min of vaping; however, these values returned to baseline after 15 min^16^.

**Table 4:** Results on measures of lung function before and after the use of an e-cigarette (or conventional cigarette, where specified). Results are shown as mean ± standard deviation.

	SpO_2_ (T0)%	SpO_2_ (T1)%	FEV_1_ T0 %pred or L or effect size [95% CI]	FEV_1_ T1 %pred or L or effect size [95% CI]	FEV_1_ T2 %pred or L or effect size [95% CI]	FEV_1_/FVC (T0) %obs or L or effect size [95% CI]	FEV_1_/FVC (T1) %obs or L or effect size [95% CI]	FEV_1_/FVC (T2) %obs or L or effect size [95% CI]	Airway Resistance (T0) kPa L^−1^ sec^−1^	Airway Resistance (T1) kPa L^−1^ sec^−1^	Airway Conductance (T0) sec^−1^ kPa^−1^	Airway Conductance (T1) sec^−1^ kPa^−1^
Palamidas 2013												
Smokers *n* = 51	-	-	-	-	-	-	-	-	0.284 ± 0.13^a^	0.308 ± 0.14^a^	1.197 ± 0.50^a^	1.060 ± 0.42^a^
Nonsmokers (11 mg§) *n* = 9	-	-	-	-	-	-	-	-	0.246 ± 0.07^a^	0.292 ± 0.05^a^	1.313 ± 0.22^a^	1.109 ± 0.18^a^
Nonsmokers (0 mg§) *n* = 10	-	-	-	-	-	-	-	-	0.247 ± 0.03^a^	0.333 ± 0.08^a^	1.213 ± 0.29^a^	0.944 ± 0.18^a^
Ferrari 2015												
Smokers *n* = 10 vs Nonsmokers CC *n* = 10	-	-	−1 [−6.1 to 4.1]	-	-	2 [−2.0 to 6.1]	-	-	-	-	-	-
Smokers *n* = 10 vs Nonsmokers EC *n* = 10	-	-	−2.1 [−5.7 to 1.6]	-	-	−0.1 [−9.8 to 3.0]	-	-	-	-	-	-
Palamidas 2017			%pred			%pred						
COPD Smokers *n* = 16	96.4 ± 1.9^a^	95.8 ± 1.8^a^	69 ± 18	-	-	59 ± 10	-	-	0.43 ± 0.18	0.47 ± 0.17	0.54 ± 0.19	0.52 ± 0.19
Asthma Smokers *n* = 11	97.2 ± 1.5	96.6 ± 1.4	93 ± 14	-	-	79 ± 11	-	-	0.38 ± 0.13^a^	0.40 ± 0.11^a^	0.84 ± 0.31	0.80 ± 0.33
Healthy Smokers *n* = 28	98.2 ± 1.0^a^	97.1 ± 1.5^a^	102 ± 15	-	-	81 ± 6	-	-	0.29 ± 0.12^a^	0.31 ± 0.13^a^	1.16 ± 0.47^a^	1.03 ± 0.40^a^
Nonsmokers (11 mg§) *n* = 9	97.4 ± 2.1	97 ± 0.7	114 ± 16	-	-	82 ± 2	-	-	0.25 ± 0.07^a^	0.29 ± 0.06^a^	1.31 ± 0.22^a^	1.11 ± 0.18^a^
Nonsmokers (0 mg§) *n* = 12	98.4 ± 0.9	98.4 ± 0.5	104 ± 10	-	-	86 ± 4	-	-	0.24 ± 0.04^a^	0.32 ± 0.08^a^	1.20 ± 0.27^a^	0.95 ± 0.18^a^
Boulay 2017			L	L		L	L					
Healthy Nonsmokers *n* = 20	97 ± 3	98 ± 1	3.9 ± 0.7	3.9 ± 0.7	-	0.80 ± 0.05	0.81 ± 0.05	-	-	-	-	-
Asthma Nonsmokers *n* = 10	98 ± 1	98 ± 1	3.4 ± 0.4	3.4 ± 0.4	-	0.78 ± 0.08	0.79 ± 0.08	-	-	-	-	-
Coppeta 2018			L	L	L	L	L	L				
Nonsmokers (CC) *n* = 30*	-	-	3.53^a,b^	3.48^a^	3.51^b^	82.2^a,b^	81.7^a^	81^b^	-	-	-	-
Nonsmokers (EC) *n* = 30*	-	-	3.55^a^	3.51^a^	3.53	82.1^a^	81.6^a^	81.5	-	-	-	-
Staudt 2018			%pred	%pred		%obs	%obs					
Nonsmokers Nicotine *n* = 7	99 ± 1	99 ± 1	112 ± 15	113 ± 11	-	81 ± 3	83 ± 3	-	-	-	-	-
Nonsmokers Non-Nicotine *n* = 3	99 ± 2	98 ± 1	103 ± 9	91 ± 8	-	81 ± 4	76 ± 4	-	-	-	-	-
Nonsmokers Total Cohort *n* = 10	99 ± 1	99 ± 1	110 ± 14	107 ± 15	-	81 ± 33	81 ± 4	-	-	-	-	-
Kizhakke Puliyakote 2021												
Nonsmokers *n* = 7	98 ± 2	-	L 3.5 ± 0.4^c^ %pred 97±8	-	-	L 0.80 ± 0.03^c^ %pred 95 ± 3^c^	-	-	-	-	-	-
Vapers *n* = 9	98 ± 1	99 ± 1	L 4.3 ± 0.9^c^ %pred 99±8	-	-	L 0.86 ± 0.04^c^ %pred 102±5^c^	-	-	-	-	-	-

*% obs* % observed, *% pred* % predicted, *CC* conventional cigarette, *CI* confidence interval, *COPD* chronic obstructive pulmonary disease, *EC* e-cigarette, *FEV1* forced expiratory volume in one second, *FEV1/FVC* ratio of FEV1 to forced vital capacity, *L* liters, *SpO*_2_ oxygen saturation, *T0* baseline (all studies), *T1* 1 min (Coppeta), 10 min (Palamidas, both studies), 60 min (Boulay), 120 min (Staudt), *T2* 15 min (Coppeta).

^a^Significant difference between T0 and T1.

^b^Significant difference between T0 and T2.

^c^Significant difference between groups at baseline.

^§^Nicotine concentration.

Airway resistance and specific airway conductance after 10 min of vaping were measured in two^14,15^ of the seven short-term studies (Table 4). Both Palamidas et al. 2013 and 2017 suggested that vaping increased airway resistance and decreased specific airway conductance among nonsmokers and smokers with and without respiratory disease. Oxygen saturation was assessed in four studies^15,17,19,20^. Three studies suggested no changes after vaping, with only Palamidas et. al. 2017 suggesting decreased oxygen saturation following vaping among smokers with and without asthma^15^.

Long-term changes (3.5 years) in lung function measurements were assessed in only one small (*n* = 21) study (Polosa 2017)^21^. This study suggested that FEV_1_, FVC, FEV_1_/FVC, and forced mid-expiratory flow (FEF_25-75_) did not change over time among vapers and non-vapers (Table 5).

**Table 5:** Prospective cohort study (Polosa 2017) on the effect of e-cigarette use on lung function over time. Results are presented as mean ± standard deviation.

	Baseline	12 ± 1 months	24 ± 2 months	48 ± 2 months
**FEV**_**1**_ **(L)**				
NonSmokers (*n* = 12)	4.08 ± 0.30	4.06 ± 0.2	4.03 ± 0.26	3.78 ± 0.71
Vapers (*n* = 9)	3.82 ± 0.78	3.81 ± 0.78	4.11 ± 0.30	3.87 ± 0.76
**FVC (L)**				
NonSmokers (*n* = 12)	5.03 ± 0.48	4.97 ± 0.42	5.01 ± 0.45	5.02 ± 0.42
Vapers (*n* = 9)	4.93 ± 0.95	4.80 ± 0.82	4.82 ± 0.91	4.87 ± 0.83
**FEV**_**1**_**/FVC (%)**				
NonSmokers (*n* = 12)	81.45 ± 5.03	82.02 ± 4.67	80.86 ± 6.18	82.06 ± 4.25
Vapers (*n* = 9)	78.49 ± 3.46	79.01 ± 3.63	78.46 ± 2.34	79.08 ± 2.83
**FEF**_**25-75**_ **(L)**				
NonSmokers (*n* = 12)	3.43 ± 0.64	3.49 ± 0.61	3.53 ± 0.57	3.56 ± 0.58
Vapers (*n* = 9)	3.29 ± 0.70	3.29 ± 0.60	3.30 ± 0.75	3.33 ± 0.64

*FEF*_25-7_ maximum mid-expiratory flow, *FEV*_1_ forced expiratory volume in 1 s, FVC forced vital capacity, L liters.

## Discussion

This systematic review was designed to determine the effect of vaping on measures of lung function. We found that there were only eight studies in the literature assessing this issue, all of which were small, and only one examined longer-term outcomes (3.5 years follow-up). In general, these studies suggest that there are no acute changes associated with vaping. However, airway resistance and conductance may be influenced by e-cigarettes, with two studies reporting changes in these values in multiple population subgroups. It is important to note that there were few studies available for this systematic review and that most of these studies focused on the acute effects of vaping; therefore, these results are suggestive but not definitive, and future research must be conducted in this area. Furthermore, three of the included studies had an unclear risk of bias, four had a moderate risk of bias, and one had a serious risk of bias, which further limits the interpretation of this review’s findings.

In addition to the limitations above, this review lacks subgroup analyses or a meta-analysis. This is due to the heterogeneity of the included studies, both in terms of study design and outcomes. Few studies were eligible for this review due to the variation in study designs and definitions of e-cigarettes and smoking status. For example, some studies included both conventional cigarette smokers and nonsmokers in their definition of “non-vapers” and did not analyze data separately based on conventional smoking status. Other studies used a “sham” vaping session for controls where either an e-cigarette with an empty cartridge (i.e., without e-liquid) or second-hand smoke were used. More commonly, studies were conducted on smokers only, without nonsmokers as a comparison group. Future studies could analyze subgroups based on both smoking and vaping status to allow for a more detailed quantitative analysis.

E-cigarettes are becoming more popular for recreational use and are being studied for harm reduction among smokers as a smoking cessation aid, as they are believed to be less harmful to health than smoking. However, there are limited data available and virtually no long-term studies assessing how prolonged e-cigarette use could impact lung function. As the use of vaping devices evolves and becomes more widespread, the health consequences of vaping are becoming an increasingly important public health issue. This is a knowledge gap that must be addressed. Knowledge of the safety of e-cigarettes, particularly their long-term safety, will inform public health policy and e-cigarette regulations, as well as the guidance clinicians, offer to their patients on smoking harm reduction. For these policies, regulations, and guidelines to be developed, we must understand how e-cigarettes can influence one’s health. This includes establishing the effects of e-cigarettes on clinical outcomes such as respiratory symptoms (cough, dyspnea), measures of lung function, and risk of developing respiratory disease. Further research is required to elucidate the short- and long-term consequences of vaping to determine whether e-cigarettes are truly a “safer” alternative to traditional cigarettes for smoking cessation or for recreational use. Future studies should be long-term, have large sample sizes, and may include different types of e-cigarettes as well as conventional cigarettes for comparison. In addition, it is important for future research to include clinical outcomes as described above. This will allow for better translation of the research findings to help inform clinical decision-making.

## Supplementary information

Supplementary Information

REPORTING SUMMARY

## Data Availability

No additional data were available, as this study is a knowledge synthesis that relied on aggregate, published results available in the public domain. Any inquiries should be directed to the corresponding author.
