# Supplementary material for: A systematic review of the effects of e-cigarette use on lung function
Source: NPJ Prim Care Respir Med. 2022 Oct 22;32:45. doi: 10.1038/s41533-022-00311-w (PMC9588082; doi:10.1038/s41533-022-00311-w)
Supplement: Supplementary file 1 — Supplementary Information [file 41533_2022_311_MOESM1_ESM.pdf]

SUPPLEMENTARY INFORMATION

Supplementary Table 1. Search strategy for studies examining the effects of e-cigarettes on lung function (MEDLINE via Ovid).

Search Number	Description
1	vaping/ or (vaping or vape*).mp. or electronic nicotine delivery systems/ or (electronic nicotine delivery or electronic cigarette* or e-cig* or ecig*).mp.
2	exp Respiratory Tract Diseases/ or exp Respiratory Function Tests/ or exp "signs and symptoms, respiratory"/ or exp Respiratory Sounds/ or exp Respiratory System/ or (airway* or breath* or bronchi* or broncho* or cardiopulmonary or chest or laryn* or lung* or mediastin* or pharyn* or pleura or pleural or pleuro* or pulmonary or respirat* or thoracic or thorax or trache* or (carbon adj (dioxide or monoxide)) or CO2 or nitric oxide or COPD or cough* or (Forced adj (Expirat* or Vital Capacit*)) or wheez* or asthma* or blood gas analys#s or capnograph* or capnometr* or oximet* or Emphysema or exhal* or inhal* or FEV1 or FVC or spiromet* or dyspnea* or Epistaxis or gas exchange* or Hemoptysis or Hypercapnia or Hyperoxia or Hyperventilat* or Hypoventilat* or Hypoxi* or sneez* or snor* or plethysmograph* or Tachypnea or ventilation or diffusion capacity or transfer factor* or (exercise adj (test* or toleran*)) or air hunger or sputum or glotti* or Supraglotti* or Epiglotti* or ((vocal cord or voice) adj (disorder* or disturbance* or dysfunction*)) or alveol* or apnea or aphonia or dysphonia or hoarse* or Pneumo*).mp. or (respirat* or lung* or pulmonary).jw
3	1 and 2
4	limit 3 to yr="2000 -Current"

Date of Search: July 12, 2021

Supplementary Table 2. Search strategy for studies examining the effects of e-cigarettes on lung function (Embase Classic + Embase via Ovid).

Search Number	Description
1	vaping/
2	electronic cigarette/
3	(vaping or vape*).mp.
4	(electronic nicotine delivery or electronic cigarette* or e-cig* or ecig*).mp.
5	1 or 2 or 3 or 4
6	exp respiratory tract disease/
7	exp lung function test/
8	common cold symptom/ or coughing/ or exp respiratory function disorder/
9	exp abnormal respiratory sound/
10	exp respiratory system/
11	(airway* or breath* or bronchi* or broncho* or cardiopulmonary or chest or laryn* or lung* or mediastin* or pharyn* or pleura or pleural or pleuro* or pulmonary or respirat* or thoracic or thorax or trache* or (carbon adj (dioxide or monoxide)) or CO2 or nitric oxide or COPD or cough* or (Forced adj (Expirat* or Vital Capacit*)) or wheez* or asthma* or blood gas analys#s or capnograph* or capnometr* or oximet* or Emphysema or exhal* or inhal* or FEV1 or FVC or spiromet* or dyspnea* or Epistaxis or gas exchange* or Hemoptysis or Hypercapnia or Hyperoxia or Hyperventilat* or Hypoventilat* or Hypoxi* or sneez* or snor* or plethysmograph* or Tachypnea or ventilation or diffusion capacity or transfer factor* or (exercise adj (test* or toleran*)) or air hunger or sputum or glotti* or Supraglotti* or Epiglotti* or ((vocal cord or voice) adj (disorder* or disturbance* or dysfunction*)) or alveol* or apnea or aphonia or dysphonia or hoarse* or Pneumo*).mp. or (respirat* or lung* or pulmonary).jw.
12	or/6-11
13	5 and 12
14	limit 13 to yr="2000 -Current"

Date of Search: July 12, 2021

Supplementary Table 3. Search strategy for studies examining the effects of e-cigarettes on lung function (PsycINFO via Ovid).

Search Number	Description
1	electronic cigarette/
2	(vaping or vape*).mp.
3	(electronic nicotine delivery or electronic cigarette* or e-cig* or ecig*).mp.
4	1 or 2 or 3
5	exp respiratory tract disorders/
6	exp respiration/
7	exp respiratory system/
8	(airway* or breath* or bronchi* or broncho* or cardiopulmonary or chest or laryn* or lung* or mediastin* or pharyn* or pleura or pleural or pleuro* or pulmonary or respirat* or thoracic or thorax or trache* or (carbon adj (dioxide or monoxide)) or CO2 or nitric oxide or COPD or cough* or (Forced adj (Expirat* or Vital Capacit*)) or wheez* or asthma* or blood gas analys#s or capnograph* or capnometr* or oximet* or Emphysema or exhal* or inhal* or FEV1 or FVC or spiromet* or dyspnea* or Epistaxis or gas exchange* or Hemoptysis or Hypercapnia or Hyperoxia or Hyperventilat* or Hypoventilat* or Hypoxi* or sneez* or snor* or plethysmograph* or Tachypnea or ventilation or diffusion capacity or transfer factor* or (exercise adj (test* or toleran*)) or air hunger or sputum or glotti* or Supraglotti* or Epiglotti* or ((vocal cord or voice) adj (disorder* or disturbance* or dysfunction*)) or alveol* or apnea or aphonia or dysphonia or hoarse* or Pneumo*).mp. or (respirat* or lung* or pulmonary).jw.
9	or/5-8
10	4 and 9
11	limit 10 to yr="2000 -Current"

Date of Search: July 12, 2021

Supplementary Table 4. Search strategy for studies examining the effects of e-cigarettes on lung function (Cochrane CENTRAL via Ovid).

Search Number	Description
1	(vaping OR vape* OR "electronic nicotine delivery" OR electronic next cigarette* OR e next cig* OR ecig*):ti,ab,kw
2	(airway* OR breath* OR bronchi* OR broncho* OR cardiopulmonary OR chest OR laryn* OR lung* OR mediastin* OR pharyn* OR pleura OR pleural OR pleuro* OR pulmonary OR respirat* OR thoracic OR thorax OR trache* OR (carbon NEAR/1 (dioxide OR monoxide)) OR CO2 OR "nitric oxide" OR COPD OR cough* OR (Forced adj (Expirat* OR Vital Capacit*)) OR wheez* OR asthma* OR blood next gas next analys*s OR capnograph* OR capnometr* OR oximet* OR Emphysema OR exhal* OR inhal* OR FEV1 OR FVC OR spiromet* OR dyspnea* OR Epistaxis OR gas next exchange* OR Hemoptysis OR Hypercapnia OR Hyperoxia OR Hyperventilat* OR Hypoventilat* OR Hypoxi* OR sneez* OR snor* OR plethysmograph* OR Tachypnea OR ventilation OR "diffusion capacity" OR transfer next factor* OR (exercise NEAR/1 (test* OR toleran*)) OR "air hunger" OR sputum OR glotti* OR Supraglotti* OR Epiglotti* OR ("vocal cord" NEAR/1 (disorder* OR disturbance* OR dysfunction*)) OR (voice NEAR/1 (disorder* OR disturbance* OR dysfunction*)) OR alveol* OR apnea OR aphonia OR dysphonia OR hoarse* OR Pneumo*):ti,ab,kw
3	#1 AND #2 with Publication Year from 2000 to 2021, in Trials

Date of Search: July 12, 2021

Supplementary Table 5. Search strategy for studies examining the effects of e-cigarettes on lung function (Web of Science Core Collection).

TS=(vaping OR vape* OR "electronic nicotine delivery" OR "electronic cigarette*" OR "e-cig*" OR ecig*) AND TS=(airway* OR breath* OR bronchi* OR broncho* OR cardiopulmonary OR chest OR laryn* OR lung* OR mediastin* OR pharyn* OR pleura OR pleural OR pleuro* OR pulmonary OR respirat* OR thoracic OR thorax OR trache* OR (carbon NEAR/1 (dioxide OR monoxide)) OR CO2 OR "nitric oxide" OR COPD OR cough* OR (Forced adj (Expirat* OR Vital Capacit*)) OR wheez* OR asthma* OR "blood gas analys*s" OR capnograph* OR capnometr* OR oximet* OR Emphysema OR exhal* OR inhal* OR FEV1 OR FVC OR spiromet* OR dyspnea* OR Epistaxis OR "gas exchange*" OR Hemoptysis OR Hypercapnia OR Hyperoxia OR Hyperventilat* OR Hypoventilat* OR Hypoxi* OR sneez* OR snor* OR plethysmograph* OR Tachypnea OR ventilation OR "diffusion capacity" OR "transfer factor*" OR (exercise NEAR/1 (test* OR toleran*)) OR "air hunger" OR sputum OR glotti* OR Supraglotti* OR Epiglotti* OR ("vocal cord" NEAR/1 (disorder* OR disturbance* OR dysfunction*)) OR (voice NEAR/1 (disorder* OR disturbance* OR dysfunction*)) OR alveol* OR apnea OR aphonia OR dysphonia OR hoarse* OR Pneumo*)
Indexes=SCI-EXPANDED, SSCI, A&HCI, CPCI-S, CPCI-SSH, BKCI-S, BKCI-SSH, ESCI, CCR-EXPANDED, IC Timespan=2000-2021

Date of Search: July 12, 2021

Supplementary Table 6. Inclusion and exclusion criteria for article screening for studies examining the effects of e-cigarettes on lung function.

Coding Manual	Detailed Description For Exclusion
1. Not relevant	It is clear the article does not address the respiratory health effects of electronic cigarette use.
2. Not original human data or primary report (e.g., reviews)	It is clear the article does not report primary data. The article is clearly either a systematic review, umbrella review, scoping review, editorial, blog post, case series, or case report. Conference and symposium abstracts containing primary data are eligible.
3. Not a randomized or non-randomized study with eligible comparators	It is clear the article is not an RCT or NRSI that evaluates the short- or long-term changes in respiratory health among individuals who vape compared to vaping abstinence.
4. No comparison group comprised of non-users of vaping devices	It is clear the article does not include non-users of vaping devices as a comparison group nor does it use a pre- and post-design where individuals act as their own controls.
5. Not measuring quantitative respiratory health outcomes relevant to this review	It is clear the article does not report quantitative outcome data on short- or long-term changes in respiratory health. Relevant outcomes include but are not limited to (1) clinical outcomes (e.g., wheeze); (2) patient-reported outcomes (e.g., shortness of breath); (3) functional outcomes (e.g., forced expiratory volume in 1 second [FEV1]); and (4) radiographical outcomes (e.g., findings of emphysema on CT scan). If it is clear the article does not contain at least one component relevant to respiratory health, it will be excluded.
